# Cross-cultural variation in understanding of animal welfare principles and animal management practices among veterinary and animal welfare professionals in the UK and Japan

**DOI:** 10.1017/awf.2025.10026

**Published:** 2025-08-06

**Authors:** Yuki Otani, Mariko Kanamori, Hiromi Kato, Cathy M. Dwyer

**Affiliations:** 1 Jeanne Marchig International Centre for Animal Welfare Education, Royal (Dick) School of Veterinary Studies, University of Edinburgh, Edinburgh, UK; 2 International Affairs Office, Faculty of Veterinary Medicine, Hokkaido University, Sapporo, Japan; 3 Social Cooperation Unit, One Health Research Centre, Hokkaido University, Sapporo, Japan; 4 Institute for the Future of Human Society, Kyoto University, Kyoto, Japan; 5 Department of Public Health Sciences, Stockholm University, Stockholm, Sweden; 6 Animal Welfare and Wildlife Damage Management Group, National Agriculture and Food Research Organisation, Tsukuba, Japan; 7 Animal Behaviour and Welfare, Department of Animal and Veterinary Sciences, Central Faculty, Scotland’s Rural College (SRUC), Edinburgh, UK

**Keywords:** Animal welfare, cultural variation, Five Freedoms, human perception, international survey, veterinary professionals

## Abstract

The World Organisation for Animal Health describes animal welfare as a “*complex and multi-faceted subject with scientific, ethical, economic, cultural, social, religious and political dimensions.*” In this study, an online survey in English and Japanese was developed based on the Five Freedoms, with the aim of investigating attitudes of veterinarians and behaviour/welfare scientists in the United Kingdom (UK) and Japan toward management of companion, farmed, experimental, zoo and wildlife animals. Respondents from the UK (n = 212) were more familiar with the Five Freedoms than those from Japan (n = 321) but both countries tended to prioritise ‘survival-related’ attributes (health and nutrition) over ‘situation-related’ attributes (behaviour) and the environmental impacts (discomfort). In Japan, however, fewer respondents recognised the ‘Freedom to express normal behaviour’ as important for domesticated animals compared to UK respondents. When considering vignettes with practical situations of cat management and dog euthanasia, UK respondents considered the provision of outdoor access to represent better management for cat welfare while most Japanese respondents thought cats should be managed entirely indoors, although the benefits and risks of going outdoors were similarly recognised in both countries. For the vignette of dog pain relating to an incurable tumour, severe pain and the dog’s mental stress motivated respondents from both countries to consider euthanasia. However, for Japanese respondents, the data suggested a perception that mental stress did not have an association with the dog’s inabilities to express normal behaviour. These data highlighted the importance of understanding the manner in which people perceive animals in different contexts and the value of considering different cultural approaches.

## Introduction

The concept of animal welfare is increasingly recognised as being of global importance in the context of managing animals. The World Organisation for Animal Health (WOAH) defines animal welfare as “*the physical and mental state of an animal in relation to the conditions in which it lives and dies.*” Animal welfare science has been applied to attempt to quantify the physical and mental state of animals, focusing on physical, physiological, behavioural and neurological evidence. In the European Union, evidence-based legislation has been developed considering the physical state of the animal, and ethological, physiological and psychological evidence concerning the animal’s behavioural motivations. WOAH also describes animal welfare as “*a complex and multi-faceted subject with scientific, ethical, economic, cultural, social, religious and political dimensions.*” This emphasises that there can be differing views as to what constitutes good animal welfare. Fraser *et al.* (1997) argues that there are at least three overlapping ethical concerns when considering the quality of animals’ lives: natural lives (which can include behaviour in the context of natural adaptations to environment); biological functioning (such as biological responses and health) and feelings (which may also include behaviours in the context of understanding emotional states); and the application or weight given to these different ethical concerns may vary across cultures and between individuals. Fraser (2008) further suggests that what people consider more or less important for animals to have good lives would be influenced by the different worldviews they have, which sometimes could lead to opposing conclusions relating to welfare issues (Fraser 2008). In this study, we explore these ideas by comparing the views of veterinarians and behaviour/welfare scientists living in two culturally distinct regions: the United Kingdom (UK) and Japan.

In the UK, animal welfare is a matter of great social concern and legislation relevant to animal protection and animal welfare has been developed. The first legislation for protecting animals in the UK was established in 1822 (Cruel Treatment of Cattle Act), which aimed to prevent cruelty towards farm animals. The impetus for the introduction of the Act was, however, more than sentimentality surrounding animal cruelty and the aim was related to improving the morals of the lower social orders rather than simply protecting animals (Radford 2001). Towards the middle of the 19^th^ Century, legislation was extended to protecting other species from suffering, with the rationale moving more towards protection from the point of view of the animals (Radford 2001). With the intensification of farming practices in the 1960s, public concern for the quality of life of animals increased and the concept of animal welfare as the Five Freedoms (FF) for animals was developed (Farm Animal Welfare Council 1993). The FF have since become the most widely referenced framework for animal welfare, conceiving of animal welfare as ‘Freedom from hunger, malnutrition and thirst’, ‘Freedom from fear and distress’, ‘Freedom from discomfort’, ‘Freedom from pain, injury and disease’ and ‘Freedom to express normal patterns of behaviour’. The FF and the Brambell Report (1965) which led to their development, specifically consider animal welfare to be related to animal feelings (hunger, thirst, pain, fear etc) and constituted a shift away from focusing on preventing animal cruelty to reflect the public desire for animals to be provided with good welfare (Broom 2011). The Animal Welfare Act (2006) expanded the species covered to include all vertebrates under human care and required owners to provide for an animal’s welfare needs and relevant Codes and Regulations offer practical standards for each species of animal. Subsequently, the Animal Welfare (Sentience) Act (2022) focused on recognising the capacity of animals to have positive and negative experiences and recognised that these capacities may also be present in certain invertebrates.

Japan is an island country in East Asia with a similarly developed economy to the UK. The first animal protection law (Act on Protection and Management of Animals [Doubutsu No Hogo Oyobi Kanri Ni Kansuru Houritsu]) was established in 1973 in response to external pressure from Western countries (Nakanishi 2016). This law has been amended every few years and was renamed the Act on Aigo and Management of animals (Doubutsu No Aigo Oyobi Kanri Ni Kansuru Houritsu; Aigo law) in 1999. The term ‘Aigo’ is composed of the characters for ‘love (愛)’ and ‘protection (護)’and its development is considered to originate from a combination of the ethics of Shinto religion, Buddhism, and Confucianism (Sato 2016). Although the definition has not reached a consensus in Japan, Aigo is generally perceived to be a subjective and emotional view held by humans wanting to protect animals in a paternalistic way (Uchikoshi 2016). One of the largest surveys concerning pet management was carried out in 2020 (Japan Pet Food Association 2020) and showed around 80% of pet owners are aware of the Aigo law, while a previous survey (taken in 2016) examining public awareness of animal welfare revealed that nearly 90% of the public had never heard of the term ‘animal welfare’ (Tokyo City University 2017). This suggests that Aigo is a predominant ethical view among Japanese when managing animals (Honjo 2022). The Aigo Law is the only legislation responsible for welfare of animals in Japan, and it applies to cattle, horses, pigs, sheep, goats, dogs, cats, domestic rabbits, chickens, domestic pigeons, domestic ducks and all other animals that are either owned or managed by humans. Although the Aigo law does not provide species-specific, evidence-based standards with legal penalties, except for dogs and cats managed for businesses such as breeding, exhibition, and training, the essence of the FF, such as ensuring an environment provides water, food, and healthcare with a consideration made for the animals’ habitat, has been included via an update in 2012 (Article 2 Fundamental Principal, Article 7 Responsibilities, etc of Owners and Possessors of Animals). The Ministry of Agriculture, Forestry and Fisheries (MAFF) has a set of newly formulated guidelines for animal management that were produced in 2023 and are based on the WOAH Terrestrial Animal Health Code for farm animals. However, neither of these guidelines are legally enforceable (MAFF 2023a).

In both the UK and Japan, most animals are similarly managed for a variety of purposes: farm, companion, experimental, zoo and free-living wildlife. Veterinarians and animal behaviour/welfare scientists play a central role in upholding and improving the welfare of those managed animals in not only practical but legal ways (Doyle *et al.* 2021). Recently, a number of veterinary schools in Japan (as well as those in the UK) have become accredited for their education by the European Association of Establishments for Veterinary Education (EAEVE), suggesting similarities in veterinary standards and training between both countries (EAEVE 2024). However, clear cultural differences in veterinary practice exist between the UK and Japan – an example of which being the attitude to euthanasia in companion animals. While in the UK an average case number for euthanasia per clinic would be 5.80 per month (Dickinson *et al.* 2014; Pegram *et al.* 2021), the average for Japan was 2.48 occasions per clinic per year (Sugita & Irimajiri 2016). Taking a broader perspective, the actual definition of animals differs between both countries. In EU and UK legislation, animals are officially defined as ‘sentient beings’ based on scientific evidence indicating that some animals have a capacity to feel and engage with the environment (Birch *et al.* 2021). Therefore, the welfare frameworks developed in those countries have been formulated to cater for animals’ needs in light of this, with a focus on what the animal feels or experiences. On the other hand, in the Aigo law of Japan, animals are defined as ‘living beings’ and proper treatment to maintain an animal’s health and safety throughout their lives is emphasised by showing respect for an animal being alive.

In this study, we hypothesised that those cultural perceptions could influence the value veterinary professionals place on good lives for animals. A questionnaire in English and Japanese was developed to investigate attitudes towards the FF as a common, global framework when managing animals. We developed two companion animal vignettes, that presented different welfare scenarios, to understand their views on approaches to managing ‘real-world’ situations. Understanding the similarities and differences regarding veterinary professionals’ perceptions of animals in different countries is key in helping develop strategies to implement good animal welfare in these diverse cultures.

## Materials and methods

### Ethical approval

The study was approved by Human Ethical Review Committee (HERC) of The Royal (Dick) School of Veterinary Studies, The University of Edinburgh, UK, approval number HERC_776_21. The survey was entirely voluntary and anonymous, and the data collected did not contain any personal and identifiable information. At the beginning of the survey, the purpose of the study was explained and the uses to which the data would be put outlined. Respondents gave informed consent to such use of these data and were made aware they could withdraw consent at any time prior to submission.

### Survey development

The survey was designed to explore participants’ understanding of the different components of animal welfare as exemplified in the FF, and to explore how important they thought these were for different groups of animals. Briefly, the initial draft of the questionnaire was created in English originally prior to being translated into Japanese by a native speaker who was also fluent in English and familiar with veterinary science and animal welfare. Questions were entirely novel and revised through several iterations among the researchers. The preliminary version of the questionnaire was piloted with a total of five volunteer veterinarians and researchers from the UK and Japan. No major changes were made from the piloted survey, however a number of minor changes were implemented in terms of numbers of a point scale (6 to 5), styles of answer (Yes/No or a point scale) and specific wordings to help ensure questions made sense in both English and Japanese.

In the first part, questions were asked relating to respondents’ demographic variables, focusing on variables known to influence animal welfare knowledge and attitudes in other studies (Serpell 2004; Hazel *et al.* 2011; Carnovale *et al.* 2024), including age, gender, profession, and previous education and training in animal welfare.

In the next part, to investigate understanding of the concept of animal welfare, respondents were asked how many of the FFs they would be able to explain to other people. Prior to proceeding to these questions, respondents were directed to look through the explanation of the FF as given by the webpage of Royal Society for the Prevention of Cruelty to Animals (RSPCA) for the UK and the Ministry of Environment (MOE) for Japan to ensure participants had equal knowledge of what the FF constitute. To investigate their perception of animal welfare, respondents were asked to select which of the FF they considered most important for companion, farmed, experimental, zoo or wildlife animals, respectively. Respondents were allowed to select multiple responses if they believed that several or all freedoms were equally important.

In the final section, respondents were shown two original fictional scenarios as ‘vignettes’: one focusing on cat husbandry practices and the other on situations in which euthanasia of a dog with incurable disease might be advised. The vignettes were carefully developed to ensure that the situation was realistic in both the UK and Japan. Questions here focused on the animal’s needs developed from the FF and relevant potential issues which might influence the animal’s welfare, including the owner’s situation with reference to relevant legislation and information from governmental bodies, veterinary associations, and animal charities. Since no metrics for measuring Japanese Aigo were included in the survey, questions relating to the individual’s love for the animal (Ai) were incorporated into the vignettes. Respondents were asked how they thought a cat owned by a man who lives in an urban area should be managed (six options, ranging from completely indoors to completely outdoors) and what they recognise as the benefits and risks to the cat when it is provided or not provided access to the outdoors. The second vignette related to a dog with an incurable femoral tumour with respondents asked whether they thought the dog should be euthanased if the situation worsened in different ways. The vignettes and related questions are described in Table 1. In this part, all questions, apart from a question regarding management preferences for the cat, were answered using a five-point Likert scale, ranging from strongly disagree to strongly agree.

**Table 1.** Detailed descriptions of two fictional scenarios (vignettes) which formed part of a survey to understand attitudes to animal welfare in Japanese (n = 321) and UK (n = 212) veterinary professionals. These vignettes formed the baseline scenario from which respondents were asked their views on how the animals should be managed

	Vignette	Following questions
Cat keeping	A man who lives alone in an urban area owns one cat (*Felis catus*). The cat is a 2 year old, neutered and microchipped female. She receives regular vaccinations (FPV, FHV, FCV, FeLV) and deworming. The owner has a single bedroom flat on the ground floor with access to the outside, surrounded by busy roads. The cat is left alone in the flat from 0800 to 2000h on weekdays. She has access to cat towers, tunnels and toys indoors. The cat is provided with adequate fresh water and food indoors and has access to two litter trays/boxes. Feral cats have been seen in the surrounding area. Please answer the following questions regarding the welfare of this cat. Please note that cats going outside should not have any effect on the security of his flat.	How would you recommend that this cat should be kept? If the cat is kept 1) indoors with access to the outdoors, or 2) always indoors do you think the living conditions allows the cat: access to proper diet and fresh water access to somewhere suitable to live to interact with other animals to express normal behaviour to be protected from illness and injury to easily be treated for illness and injury to avoid conflict with humans to avoid becoming lost to be deeply loved by the owner
Dog euthanasia	A 9 year old neutered male border collie (*Canis lupus familiaris*) has been diagnosed with a tumour on his femur, for which there is no hope of a cure. The owner is keen to give the dog every possible treatment at home. The dog’s appetite has decreased to about 50% and the pain is severe, but can be controlled with painkillers at the moment The dog is no longer able to run but can walk slowly and still appears to enjoy going outside for a walk. He is able to defaecate and urinate on his own.	Should the dog be euthanased at this point? Under which following conditions would you consider that the dog should be euthanased? When severe pain can no longer be managed by any medication When the mental stress of the dog becomes severe When he is no longer able to intake water or food by himself When he is no longer able to control defaecation and urination When the dog is no longer able to walk by himself When the owner’s situation worsens When the owner physically can no longer care for the dog by himself/herself When the owner no longer loves the dog When the dog becomes too much trouble for the owner or other humans (e.g. bite, bark) When the owner asks vet to euthanase the dog

FPV: Feline Panleukopenia Virus, FHV: Feline Herpesvirus, FCV: Feline Calicivirus, FeLV: Feline Leukemia Virus.

Google Form was used as a platform for the survey to make it accessible for respondents in both countries and the survey can be seen in Supplementary material_1.

### Respondents

The study target sample population consisted of veterinarians, and animal behaviour and welfare scientists who were working in either the UK or Japan. Participants were recruited via a variety of means, including emails sent to national and international associations related to veterinary medicine, farm animals, zoo, racehorses, animal behaviour and welfare. Further, recruitment was advertised on the webpage, and social media (Facebook and X [formerly Twitter]) of the Jeanne Marchig International Centre for Animal Welfare Education at the Royal (Dick) School of Veterinary Studies in Edinburgh, UK, and we also called for participants at a Royal Canin Veterinary Symposium in Japan and in *The Veterinary Record* of the British Veterinary Association. The survey was open from 11^th^ November 2021 to 31^st^ January 2022 with respondents divided into either the UK or Japan depending upon where they were currently working. In total, 215 and 363 respondents from, respectively, the UK and Japan were recruited.

### Statistical analysis

Responses from respondents who were not in the target population (including veterinary nurses and students), and those for whom eligibility was unclear, were excluded from the analysis. The final analysis, therefore, included data from 212 and 321 respondents from UK and Japan, respectively. To facilitate analysis, age categories were regrouped into four categories (< 35, 35–44, 45–54, > 54 years) from 6 (< 25, 25–34, 35–44, 45–54, 55–64, > 65 years old) to deal with low numbers in the extreme age classes. Similarly, categories of profession were regrouped to provide three classes for analysis (veterinarian in practice, veterinary researcher, animal behaviour/welfare researcher) from seven original options (veterinarian in practice, veterinary researcher, veterinarian in public health, veterinarian in another role, researcher of animal behaviour, researcher of animal welfare, other) in order to make them sufficiently balanced. The data were entered into Microsoft Excel® (Microsoft 365) and all analyses performed using the statistical software STATA 16.1 (StataCorp, TX, USA). The answers obtained via Likert scales were converted to a number (strongly disagree: 1 to strongly agree: 5) and then used for statistical analysis. In the section considering cat management, respondents were asked to evaluate if the cat in the vignette would have access to factors relating to the FF (food, water, proper environment, interaction, treatment etc) if management included access to the outdoors or not (on a five-point Likert scale). The view of the respondents regarding the relative benefits or risks of being outdoors were then calculated as the difference in responses given in relation to the cat being completely indoors. Then, the answers were converted into numbers, with the values of answers when completely indoors subtracted from values for when the cat has access to outdoors, in order to evaluate the relative benefit or risks recognised by respondents. Chi-squared or Mann-Whitney *U* tests were used to compare responses across countries. Knowledge and attitude towards the relative importance of the FF within each country were investigated using Binary Logistic regression fitting age, profession and education as fixed effects. Binary Logistic Regression for familiarity with the FF was conducted by dividing responses into two groups (those who felt they could explain 0–3 = 0, those who were more confident in being able to explain 4–5 = 1) to investigate predictors of this familiarity. Factor Analysis with promax rotation was performed on the data investigating respondents’ attitudes to euthanasia of the dog in response to different scenarios (Table 1) to investigate relationships of variables relevant to an animal and owner when discussing potential euthanasia of the dog.

## Results

### Respondent demographics

The distribution of respondents between different categories is shown in Table 2. In both countries, respondents were predominantly practising veterinarians (approximately two-thirds of respondents in both countries) and around 90% of all respondents were veterinarians including researchers or in another role. The gender distribution was, however, significantly different with more than 85% of respondents from the UK being women whereas 57% of respondents from Japan were men (χ^2^[2] = 110.1; *P* < 0.001). The UK participants were skewed towards the younger demographic with more than half of respondents younger than 35 years old and less than 20% of respondents older than 45 years old. For the Japanese respondents, ages were more evenly distributed across age categories (χ^2^[3] = 41.9; *P* < 0.001).

**Table 2.** Demographic information of 533 veterinary professional respondents who took part in a survey of attitudes to animal welfare from UK (n = 212) and Japan (n = 321). The number of respondents in each category are presented with the proportion of the total shown as a percentage in parenthesis

	UK	Japan
**Profession**		
Vet in practice	142 (67.0)	200 (62.3)
Veterinary/biological researchers and vet in other role	44 (20.8)	96 (29.9)
Animal behaviour/welfare researcher	26 (12.3)	25 (7.79)
**Gender**		
Female	184 (86.8)	133 (41.4)
Male	26 (12.3)	184 (57.3)
Prefer not to say	2 (0.9)	4 (1.3)
**Age**		
< 35	114 (53.8)	91 (28.3)
35–44	57 (26.9)	99 (30.8)
45–54	18 (8.5)	74 (23.1)
> 55	23 (10.8)	57 (17.8)
**Formal education in animal welfare**	199 (93.9)	181 (56.4)
**Self education in animal welfare**	144 (67.9)	214 (66.7)

In the comparison of educational background, 94% of UK respondents answered they had received a formal education in animal welfare compared to 56% of Japan respondents (χ^2^[1] = 87.6; *P* < 0.001). The effects of age and profession were assessed using Binary Logistical Regression (Table S1; Supplementary material_2) to investigate predictors of education in animal welfare. Despite there being no significant effects of age or profession on the likelihood of receiving formal education in animal welfare in the UK, in Japan respondents under the age of 35 years were significantly more likely to have received formal education in animal welfare compared to older respondents (*P* < 0.001). For self-education in animal welfare, equivalent numbers of UK and Japanese respondents (67.9 and 66.7%, respectively) had sought to find animal welfare education themselves as well as or instead of receiving it as part of their formal education (χ2[1] = 0.0916; *P* = 0.762). In both countries this was more prevalent in animal welfare researchers than in veterinarians, whether they worked in practice or in research (Table S1; Supplementary material_2).

### Attitude towards the Five Freedoms

**Figure 1.**
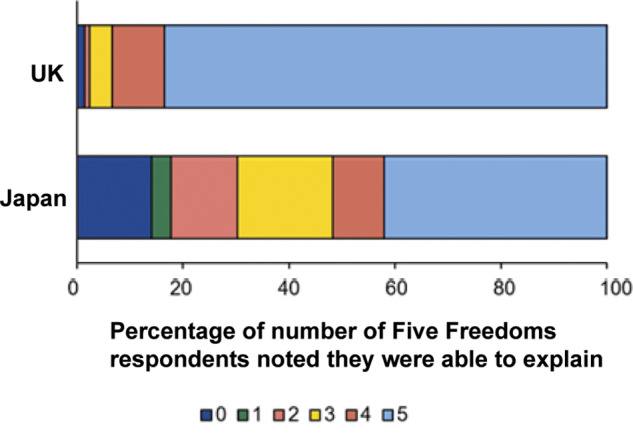
The percentage of UK (n = 212) and Japanese (n = 321) respondents in a survey of attitudes to animal welfare who considered themselves able to explain 0, 1, 2, 3, 4 or 5 of the Five Freedoms to others.

To investigate how familiar respondents were with the FF, they were asked how many they could explain to others. Most (83.5%) respondents from the UK considered themselves able to explain all five (Figure 1). In Japan, however, 51.7% of respondents were of the opinion they could explain four or five but nearly 20% responded that they could explain none or one only, resulting in a significant difference between the two countries (χ2[5] = 108.8; *P* < 0.001). For both countries, seeking self-education in animal welfare was associated with a greater likelihood of the belief of being able to explain four or five freedoms (Table 3). Further, in Japan, younger respondents were more confident they could explain four or five freedoms than older respondents. Similarly, animal behaviour/welfare scientists considered themselves more able to explain four or five freedoms than did the veterinarians.

**Table 3.** Result of binary logistic regression using demographic data to investigate predictors of the number of the Five Freedoms respondents can explain to others in the UK (n = 212) and Japan (n = 321) (Original responses are scaled as 0-3=0, 4-5=1). In both countries, respondents who undertook self-education were able to explain more Freedoms whereas age and profession were also associated for Japanese respondents

	UK			JAPAN		
	OR	*P*	[95% CI]	OR	*P*	[95% CI]
**Age (ref. <35)**						
35–44	0.64	0.53	[0.16, 2.55]	0.42	0.008	[0.22, 0.78]
45–54	0.43	0.37	[0.07, 2.74]	0.45	0.037	[0.21, 0.95]
> 55	0.77	0.82	[0.07, 8.04]	0.88	0.76	[0.37, 2.06]
**Profession (ref. Vet in practice)**						
Veterinary researcher	0.84	0.82	[0.19, 3.76]	1.8	0.034	[1.04, 3.09]
Animal behaviour/welfare researcher	0.89	0.92	[0.09, 8.49]	7.58	0.002	[2.09, 27.47]
**Formal education (ref. No)**	4.1	0.077	[0.86, 19.61]	1.54	0.15	[0.85, 2.78]
**Self education (ref. No)**	6.6	0.005	[1.76, 25.19]	3.81	<0.001	[2.24, 6.48]

OR: Odds Ratio; CI: Confidence Interval.

**Figure 2.**
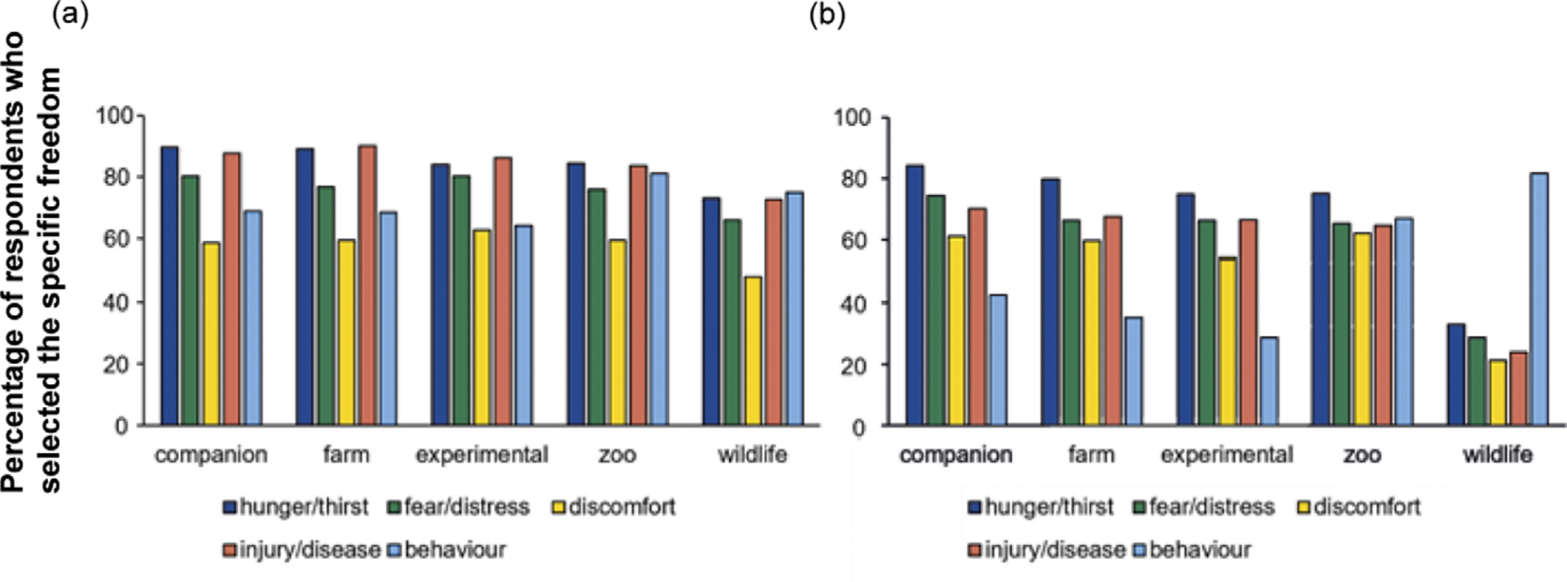
Histogram showing (a) UK (n = 212) and (b) Japanese (n = 321) respondents’ responses when asked to choose which of the Five Freedoms was most important for companion, farm, experimental, zoo or wildlife animals. Each bar shows the percentage of respondents who selected the freedom in question as most important. Respondents were able to select 1 to 5 freedoms for each category of animals. Key: Hunger/thirst: Freedom from hunger, malnutrition and thirst; Fear/distress: Freedom from fear and distress; Discomfort: Freedom from discomfort; Injury/disease: Freedom from pain, injury and disease; Behaviour: Freedom to express normal patterns of behaviour.

We next investigated which freedoms respondents considered the most important for animals in each category (Figure 2). In the UK, respondents selected ‘Freedom from hunger and thirst’ and ‘Freedom from injury, pain and disease’ most frequently for all categories of animals, although at a lower rate for wildlife (companion 89.6, 87.7%; farm 89.2, 90.1%; experimental 83.4, 86.3%; zoo 84.4, 89.5%; wild 73.1, 72.6%, respectively). ‘Freedom from fear and distress’ was selected at a lower rate but still considered important by most respondents in the UK (companion 80.2%; farm 76.9%; experimental 80.2%; zoo 76.0%; wild 66.0%). UK respondents consistently rated ‘Freedom from discomfort’ as the least important freedom, in all categories (companion 58.5%; farm 59.4%; experimental 62.7%; zoo 59.4%; wildlife 48.1%). ‘Freedom to express normal behaviours’ was also considered to be less important than other freedoms for companion, farm and experimental animals, but to be comparable with ‘Freedom from hunger and thirst’, and ‘Freedom from injury, pain and disease’ for zoo and wildlife welfare (companion 68.9%; farm 68.4%; experimental 64.2%; zoo 81.1%; wildlife 75.0%).

For Japanese respondents, ‘Freedom from hunger and thirst’ was also consistently selected as the most important except for wildlife (companion 84.1%; farm 79.8%; experimental 65.1%; zoo 75.1%; wildlife 33.0%). The next most commonly prioritised freedoms were ‘Freedom from fear and distress’ and ‘Freedom from injury, pain and disease’ except for wildlife (companion 74.5, 70.1%; farm 66.0, 67.3%, experimental 54.2, 66.4%; zoo 65.1, 64.5%, wildlife 28.7, 24.0%, respectively). Similarly to the UK, ‘Freedom from discomfort’ was selected less frequently in Japan (companion 61.1%; farm 59.5%; experimental 54.2%; zoo 62.0%; wildlife 21.2%). However, ‘Freedom to express normal behaviour’ was less likely to be selected as important by Japanese respondents for companion, farm and experimental animals, although it was ranked equivalent to other freedoms for zoo animals and was the highest ranked freedom for wildlife (companion 42.4%; farm 35.2%; experimental 28.7%; zoo 66.7%; wildlife 81.6%). These attributes resulted in a variety of the numbers of freedoms that respondents selected among animal category within country and also between countries (Figure S1; Supplementary material_2; UK vs Japan: companion 3.85 vs 3.32; farm 3.84 vs 3.08; experimental 3.77 vs 2.90; zoo 3.84 vs 3.33; wildlife 3.35 vs 1.88; *P* < 0.001, Mann-Whitney *U* test, n = 212 [UK]; 321 [Japan]). In particular, Chi-squared tests confirmed that ‘Freedom from injury and disease’ and ‘Freedom to express normal behaviour’ were less frequently selected in Japan compared to the UK, regardless of categories apart from wildlife (Table S2; Supplementary material_2).

To further investigate factors influencing differences between countries, we focused on the data for companion animals and Binary Logistic Analysis was performed on the results of ‘Freedom from injury and disease’ and ‘Freedom to express normal behaviour’ using demographic data (Table 4). There was no effect of age, profession or education in animal welfare on the probability of selecting ‘Freedom from injury and disease’ as important in either the UK or Japan. This was also true for ‘Freedom to express normal behaviour’ for Japanese respondents. However, in the UK, those respondents who had invested in self-education in animal welfare were more likely to consider ‘Freedom to express normal behaviour’ as important (Odds Ratio: 3.00; [95% Confidence Interval: 1.55, 5.82]; *P* = 0.001).

**Table 4.** Result of binary logistic regression using demographic data to investigate predictors for selecting ‘Freedom from injury and disease’ and ‘Freedom to express normal behaviour’ as the most important (or equally most important to others) of the Five Freedoms from respondents in the UK (n = 212) and Japan (n = 321)

	Freedom from injury and disease						Freedom to express normal behaviour					
	UK			JAPAN			UK			JAPAN		
	OR	*P*	[95% CI]	OR	*P*	[95% CI]	OR	*P*	[95% CI]	OR	*P*	[95% CI]
**Age (ref. <35)**												
35–44	0.87	0.78	[0.31, 2.42]	0.80	0.50	[0.43, 1.51]	1.35	0.42	[0.65, 2.80]	1.07	0.82	[0.59, 1.94]
45–54	2.27	0.45	[0.26, 19.48]	0.90	0.79	[1.43, 1.89]	10.05	0.031	[1.24, 81.40]	1.33	0.42	[0.66, 2.66]
> 55	0.33	0.087	[0.092, 1.17]	1.24	0.62	[0.53, 2.89]	1.39	0.58	[0.43, 4.54]	1.97	0.081	[0.92, 4.23]
**Profession (ref. Vet in practice)**												
Veterinary researcher	0.72	0.56	[0.24, 2.16]	1.17	0.58	[0.67, 2.04]	1.51	0.37	[0.61, 3.72]	0.92	0.75	[0.55, 1.52]
Animal behaviour/welfare researcher	0.38	0.12	[0.12, 1.27]	0.74	0.51	[0.30, 1.82]	0.69	0.46	[0.26, 1.83]	1.62	0.27	[0.68, 3.86]
**Formal Education (ref. No)**	1.30	0.75	[0.25, 6.62]	0.98	0.93	[0.55, 1.73]	1.56	0.51	[0.42, 5.82]	1.25	0.42	[0.73, 2.14]
**Self Education (ref. No)**	1.58	0.35	[0.61, 4.08]	1.06	0.83	[0.63, 1.78]	3.00	0.001	[1.55, 5.82]	1.05	0.84	[0.64, 1.71]

OR: Odds Ratio; CI: Confidence Interval

### Vignette 1: Opinions on cat keeping

**Figure 3.**
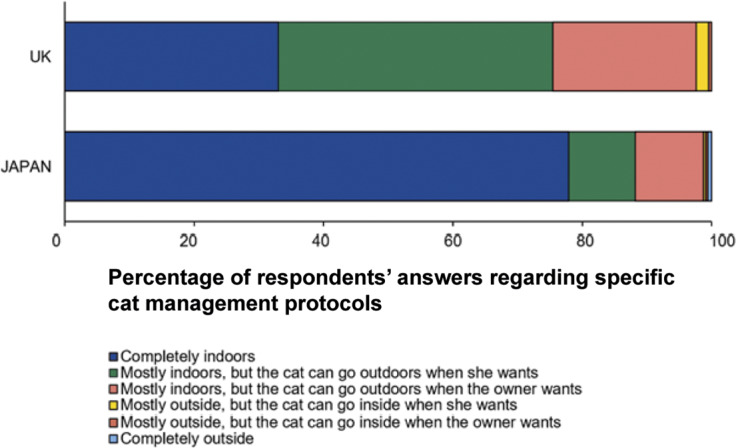
Bar chart showing the percentage of UK (n = 212) and Japanese (n = 321) respondents’ answers when presented with a series of management options relating to the scenario of a city-dwelling owner with a single cat. Respondents were allowed to select 1 of 6 management categories that differed in terms of whether the cat was indoor, outdoor, or both; and whether the decision was made by the cat or the owner.

**Figure 4.**
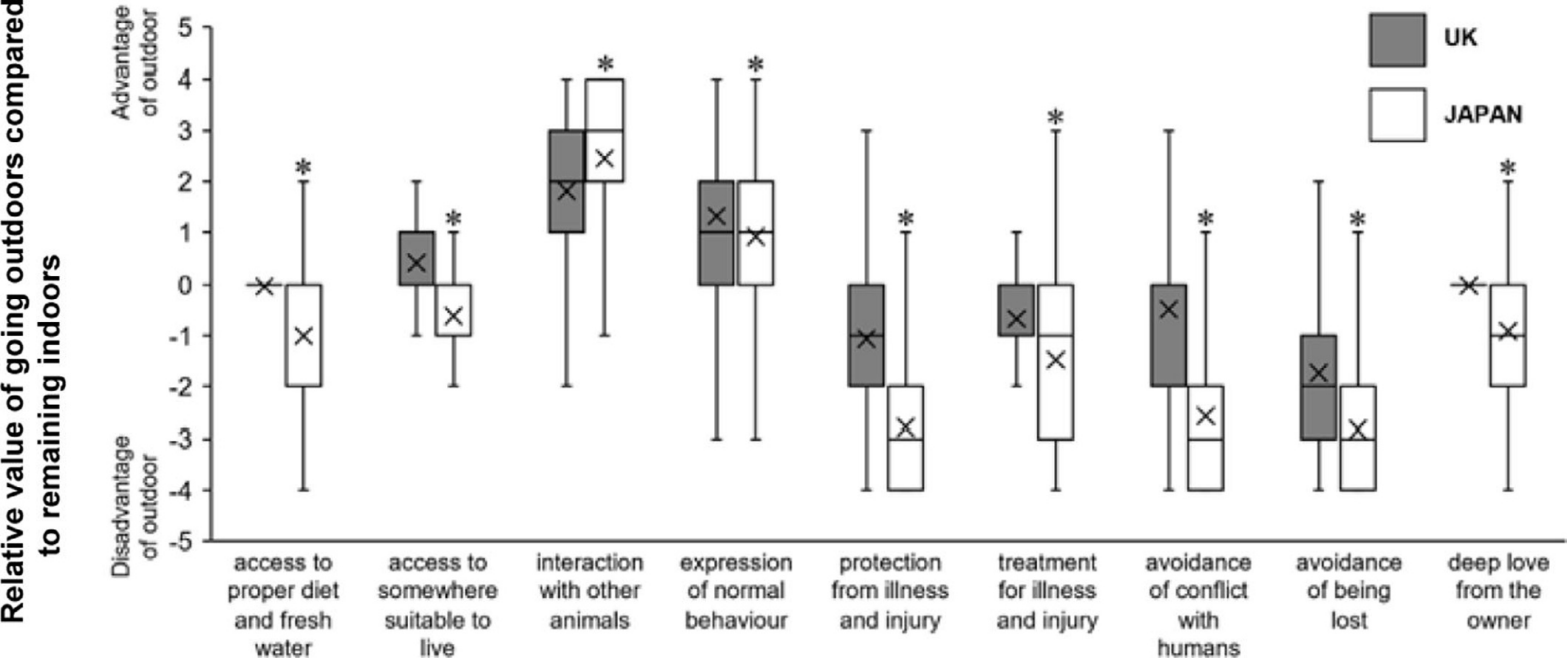
Box and whisker plot of the relative value of a cat going outdoors in relation to relevant factors as selected by respondents as the benefit or risk factors for cat management, in comparison to staying completely indoors. The relative value was calculated by subtracting the answers when the cat was managed completely indoors from the answers when access to outdoors was provided. The box and whisker plot represents the minimum, first quartile, median, third quartile, and maximum. Cross marks represent an average, and asterisks show significant differences between the UK (n = 212) and Japan (n = 321). *P < 0.05, Mann-Whitney *U* test.

When asked how the cat should be managed, although both groups of respondents considered that cats should spend most of their time indoors (more than 95% of responses; Figure 3), respondents from the UK were much less likely to consider the cat should be kept completely indoors compared to those from Japan (UK 33.0%; Japan 77.9%; χ2[5] = 116.12; *P* < 0.001; Figure 4). UK respondents were most likely to choose: ‘mostly indoors, but the cat can go outdoors when she wants’ (42.5%) and ‘mostly indoors, but the cat can go outdoors when the owner wants’ (22.2%) rather than keeping the cat only indoors.

We next investigate perceptions of respondents toward going outdoors and staying indoors in order to understand what were deemed to be the benefits and risks for the cat. Figure 4 shows the relative value of the cat being allowed access to outdoors compared to being kept entirely indoors for different attributes based on the FF and Aigo concepts. Overall, respondents from both countries were in broad agreement regarding their assessment of the risks and benefits of allowing the cat access to outdoors access, i.e. that such access could provide positive experiences to help express normal behaviour while risks relating to injury, becoming lost, and having conflicts with humans became elevated (Figure 4).

However, Japanese respondents were more likely than their UK counterparts to view those risks more negatively for the cat (*P* < 0.01, Mann-Whitney *U* test). In addition, Japanese respondents considered outdoor access to negatively impact access to food, water and a comfortable environment, whereas UK respondents perceived it to be neutral for food and water access and relatively positive for the environment. Further, Japanese respondents also considered that allowing a cat access to outdoors reduced the love or affection the owner had for the cat while UK respondents considered this to be neutral.

### Vignette 2: Opinions on dog euthanasia

**Figure 5.**
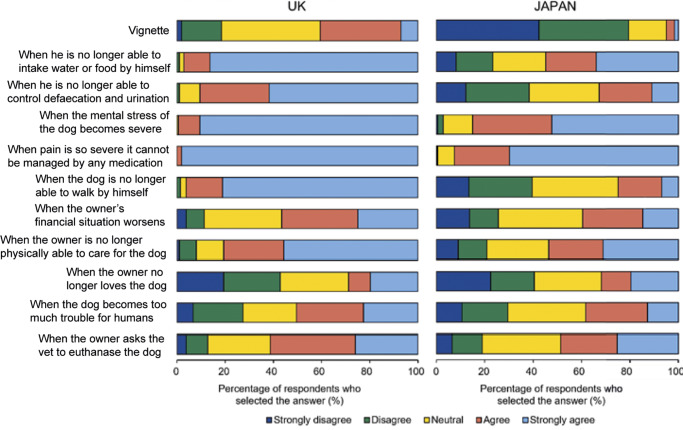
Percentage of UK (n = 212) and Japanese (n = 321) respondents who selected Strongly disagree, Disagree, Neutral, Agree or Strongly agree to euthanase a dog when presented with the scenario of a nine year old dog with an incurable femoral tumour. In addition to the baseline scenario, altered dog and owner scenarios were also presented (as shown on the left of the graph).

When considering the second vignette of a dog euthanasia case, 5.0% of respondents from Japan supported euthanasia for the dog as presented in the base scenario, whereas 40.6% of UK respondents agreed that euthanasia at that point should be performed (Figure 5). Furthermore, 79.4% of Japanese respondents either disagreed or strongly disagreed with euthanasia for the dog at this point while only 18.4% of UK respondents were opposed (χ2[2] = 104.5; *P* < 0.001). These opinions were further investigated in light of alterations to the status of the dog and the owner. For both the UK and Japan, all changes (with the exception of the owner’s love) increased the percentages of agreement (strongly agree and agree) and UK respondents were consistently more likely than the Japanese to support euthanasia in all the conditions (*P* < 0.05, Mann-Whitney *U* test). All the respondents from UK (100%) and virtually all from Japan (92.5%) expressed agreement for euthanasia when severe pain was no longer able be managed by any medication. Both countries also showed another large increase in the scenario whereby the dog’s mental stress became severe, although for Japanese respondents the increase was not as large as seen for the dog’s uncontrollable pain (UK 99.5%; Japan 85.1%). For UK respondents, the proportion agreeing euthanasia was required was also comparable for the scenario whereby the dog was no longer physically able to take in food or water of its own accord (97.2%), or able to control defaecation (90.6%) or able to walk unaided (96.2%). On the other hand, in Japan, 54.8, 32.7, and 24.9% of respondents, respectively, expressed a positive attitude towards the euthanasia under those respective situations and approximately 40% expressed opposition to euthanasia in situations of uncontrolled defaecation or inability to walk. When conditions were suggested relating to the owners’ requests or abilities to take care of the dog, similar responses between both Japanese and UK respondents were observed.

In the Factor Analysis (Table 5), for both the UK and Japan, ten variables associated with the dog or owner’s condition were grouped similarly into three dimensions. The first factor identified was characterised by five variables relating to the views of, or interaction with, the owner (factor variance/proportion: UK 2.825/0.717; Japan 2.944/0.636). The second factor was characterised by variables related to the dog’s inabilities to express self-directive behaviours, including eating, drinking, defaecating, and walking (factor variance/proportion: UK 1.742/0.442; Japan 2.459/0.532). The third factor characterised the dog’s mental stress and pain (factor variance/proportion: UK 1.052/0.267; Japan 2.120/0.458). For Japanese respondents, all variables clearly showed the highest factor loadings (more than 0.6 except for two variables) in one of three factors. On the other hand, in the UK, factor loadings of variables in Factors 2 and 3 were lower than those of the Japanese respondents, and dog’s mental health contributed to both Factor 2 (0.34) and 3 (0.39), while in Japan it contributed only to Factor 3 with a higher loading (0.73).

**Table 5.** Result of factor analysis of respondents’ responses to situations asking when they would or would not advocate euthanasia of a dog with an incurable tumour in the UK (n = 212) and Japan (n = 321). Variables were grouped similarly into three dimensions in both countries. actors with the owner as Factor 1; the dog’s inabilities to express self-directive behaviours as Factor 2; the dog’s mental stress and pain as Factor 3

	UK				JAPAN			
	Factor1	Factor2	Factor3	Uniqueness	Factor1	Factor2	Factor3	Uniqueness
When severe pain can no longer be managed by any medication	0.01	0.12	**0.51**	0.68	0.07	–0.02	**0.73**	0.44
When the mental stress of the dog becomes severe	0.02	**0.34**	**0.39**	0.62	–0.04	0.06	**0.73**	0.44
When he is no longer able to intake water or food by himself	–0.12	**0.66**	0.17	0.49	–0.02	**0.55**	0.22	0.52
When he is no longer able to control defaecation and urination	0.07	**0.61**	–0.17	0.65	–0.01	**0.79**	0.02	0.36
When the dog is no longer able to walk by himself	0.05	**0.43**	0.13	0.74	0.04	**0.74**	–0.07	0.48
When the owner’s financial situation worsens	**0.60**	0.24	–0.01	0.50	**0.71**	0.12	–0.01	0.41
When the owner physically can no longer care for the dog by himself/herself	**0.68**	–0.06	0.13	0.51	**0.80**	0.01	0.03	0.33
When the owner no longer loves the dog	**0.78**	0.01	–0.08	0.42	**0.82**	–0.11	–0.02	0.40
When the dog becomes too much trouble for the owner or other humans (e.g. bite, bark)	**0.79**	0.01	–0.02	0.40	**0.61**	0.01	0.04	0.61
When the owner asks vet to euthanase the dog	**0.75**	0.11	0.05	0.46	**0.54**	0.04	0.00	0.69

## Discussion

In this study, the attitude of veterinarians and animal behaviour/welfare scientists from the UK and Japan towards animal welfare and animal management were investigated. Most of the participants from both countries were veterinarians however there was a significant difference in the gender ratio. Since most of the UK respondents were under 45, this ratio approximately reflects the current population demographics for veterinarians in each country (MAFF 2023b; IES 2024). We found that respondents from the UK were more familiar with the concept of the FF than those from Japan and considered themselves more confident in explaining these but younger respondents in Japan were more likely to have received some formal education and knowledge in animal welfare. Almost two-thirds of respondents in both countries had sought out education in animal welfare themselves and this contributed to improving their knowledge on the FF compared to undertaking passive education. When asked to nominate the most important freedom(s) for each category of animal, respondents from Japan selected a significantly smaller number of freedoms than those from the UK. Taking the two vignettes, Japanese respondents were more likely than those from the UK to consider that cats should be managed indoors for the entirety of their lives, although the benefits and risks of the cat being outdoors were similarly recognised by both. While 40% of UK respondents considered euthanasia to be the best option for the dog in this vignette, approximately 80% of respondents in Japan expressed their clear opposition to such an outcome. Severe pain and mental stress for the dog were most likely to motivate respondents from both countries to consider euthanasia. However, the association between mental stress and the dog’s inabilities to drink water, eat food, defaecate and walk was recognised in respondents from the UK, not Japan.

Despite those differences both countries prioritise ‘survival-related’ attributes for welfare (health and nutrition) over ‘situation-related’ attributes (behavioural interactions; Mellor *et al.* 2020), although the environmental impacts (discomfort) were also not considered the most important. This may have been a direct result of the majority of participants being veterinarians. For both countries, the respective veterinary curricula focus upon animals’ physical health, with less attention paid to behavioural responses, leading veterinarians to sometimes present the view that health is the core part of welfare (Broom 2011). On the other hand, respondents from both countries considered behaviour to be more important for the welfare of wild animals, whether captive or free-ranging. Neither the UK nor Japan extends animal welfare legislation to include free-living wildlife, since it focuses on animals under human control for which a perceived duty of care exists to consider an animal’s physical and mental needs. Wildlife may be considered well adapted to their environments and thus able to access food for as long as they are able to roam and forage. Further, fear and injury are perhaps considered to be reasonably inevitable in their wild habitat. Alternatively, or additionally, the emphasis on behaviour only for wildlife may be associated with an assumption that domesticated animals have less need to show behavioural adaptations and may be less motivated to show specific behaviours. This is not supported by research demonstrating domesticated animals to still continue to show highly motivated behaviours in captivity (e.g. pigs [*Sus scrofa*]: Stolba & Wood-Gush 1984), and prevention of these can cause behavioural frustration and physiological indicators of stress (for a review, see Mason & Burn 2018). Many of the welfare issues facing farm, experimental and companion animals, such as stereotypy, tail-biting, and destructive behaviours, stem from preventing the expression of highly motivated components of the animal’s behavioural repertoire (e.g. Bradshaw *et al.* 2002; D’Eath *et al.* 2014; Roberts *et al.* 2017). These data suggest that respondents from both countries potentially undervalue the impact of behaviour-related welfare issues in favour of more immediate or possibly short-term physical issues. Poor health or a lack of suitable nutrition are clearly very important for the immediate survival of an animal, and it may be that participants considered these ‘survival-critical’ factors in their consideration of the importance of the different factors, rather than taking into account the chronic or ongoing welfare impacts that behavioural restriction can cause. As UK respondents who had sought to find animal welfare education themselves improved their knowledge, education should help people become aware of the importance of behavioural interactions.

There was also a country-specific impact whereby less than 40% of respondents from Japan classified ‘Freedom to express normal behaviour’ as being important for companion, farm and experimental animals compared to around 70% for UK respondents. This was not explained by any demographic background variables suggesting that some contextual characteristics in Japan might contribute to the attitude. It is possible that the Japanese interpretation of how humans should treat animals (based on Aigo), which focuses more on ‘protection’ of animals, has led to a greater focus on survival-related attributes, while in the UK, a history of considering animal experience and how behaviour can be both a tool in this assessment (Dawkins 2003, 2004), and an important component (Fraser 2008; Hemsworth *et al.* 2015) predominates. A marked increase of the importance of the ‘Freedom to express normal behaviour’ for wildlife could be affected by religious and cultural norms in each country (Yeshey *et al.* 2024). Practically, for instance, the Japanese government officially discourages citizens from feeding wildlife from the viewpoint of respecting the interdependence of wildlife as part of ecology, the potential risk of conflict between animals and humans, and infectious disease (MOE 2021), all of which might mirror Japanese traditional attitudes toward wildlife. On the other hand, in the UK, a survey by the RSPCA (2022) reported that people considered feeding wild birds to be ethically good, suggesting human intervention, at least for wild birds, may be more accepted as an action to improve their welfare.

Our data suggest that both countries’ respondents altered their attitude depending on the category of animal, with the tendency more pronounced in the Japanese. Uchikoshi (2016) described this disparity in Japan as a “*compartmentalised view on animals*” and hypothesised that it may be partially explained as people’s concern/interest and academic discipline being compartmentalised depending on the categories into which animals are divided. This unconscious/conscious compartmentalisation might be applied in other countries, including the UK, to a different extent or to different species. In the UK, however, sentient animals, that is those capable of experiencing positive and negative emotions, have been identified as all vertebrates, decapod crustaceans and cephalopods in the Animal Welfare (Sentience) Act 2022. This legal definition of animal sentience might encourage respondents to be aware that these animals should be afforded an equally good quality of life as sentient beings, regardless of their purpose or use by humans and thereby minimise the compartmentalisation.

The use of vignettes describing animal management helped us further investigate how attitudes towards different aspects of welfare translated into preferred actions. In responses to both vignettes, the rather subtle differences in attitude towards the FF in our UK and Japanese respondents resulted in quite marked differences in practices towards animal management. Questions regarding the cat highlighted a clear difference in terms of whether cats should be allowed access to the outdoors, with only 20% of respondents from Japan suggesting the cat should be allowed to go outside compared to over 60% of UK respondents. Interestingly, although all respondents broadly identified the same risks and benefits to welfare in terms of indoor and outdoor management, Japanese respondents tended to estimate the disadvantages more negatively than those from the UK, particularly the risk of injury, conflict with humans and becoming lost. Hofstede (2011) proposed six dimensions of national cultures and one of the most remarkable gaps between the UK and Japan was ‘Uncertain avoidance’ (UK score 35; Japan score 92) (Hofstede 2011; Taniguchi *et al.* 2022). This might explain that Japanese respondents viewed the possible lack of control and potential risks the cat may face outdoors more negatively and would thereby seek to avoid these situations more keenly than those from the UK.

These preferences may also be influenced by each country’s social norms, and the relevant legislation and professional training they will have received. In Japan, Standards relating to the Care and Keeping Animals at Home, which is based on the Aigo law, describes that owners of cats should make efforts to keep cats completely indoors (MOE 2013). Government advertisements stress the potential risks of infection, road traffic accidents, being lost, unpredictable pregnancy, and aggressive encounters with other cats (MOE 2014a). Consequently, numbers of completely indoor cats are on the increase and 80% of owned cats in Japan were reportedly kept completely indoors in 2022 (Japan Pet Food Association 2022). In the UK, on the other hand, there is no veterinary consensus regarding indoor or outdoor keeping of cats (Yeates & Yates 2017), but the People’s Dispensary for Sick Animals (PDSA) PAW report (2024a) suggests that 68% of UK cats have a degree of outdoor access. The Department for Environment, Food and Rural Affairs (DEFRA) makes no recommendation regarding whether cats should live indoors or outdoors in their Code of Practice for the welfare of cats (DEFRA 2017) but they do emphasise the need to provide opportunities for play and exercise indoors if the cat does not go outside. Furthermore, some UK charities, such as Cats Protection and the PDSA describe providing a cat outdoor access as being crucial for their mental health, to provide a natural life and to avoid obesity and stress (Cats Protection 2015; PDSA 2024b). However, these charities, amongst others, also provide information for promotion of natural behaviour in indoor-only cats, giving due consideration to the risks posed by disease, traffic, and potentially becoming lost.

Many countries actively debate the issue of whether or not cats should be allowed to free-roam outside, with the USA, Canada and Australia advocating for increased indoor management of cats, while Europe and New Zealand more commonly sees a mixed indoor/outdoor lifestyle (Foreman-Worsley & Farnworth 2019; Tan *et al.* 2020). However, not all these decisions are predicated on cat welfare, and may be impacted more by risks of predation of native wildlife by outdoor cats, an issue which extends beyond the scope of our study. It is striking, however, that in the UK the benefits of natural behaviour and reduced stress associated with outdoor access are typically emphasised, whereas in Japan it is the negative consequences of injury, disease and conflict that are most likely to be cited. As Aigo represents a subjective and emotional perception there may be no subjective scale to evaluate it. However, the amount of time and effort expended by the owner would be relevant to Aigo ethics, since Japanese respondents consideration of indoor keeping involved complete control over food, water, and the cat’s environment by the owner. Moreover, this closer and more intimate physical relationship encapsulates the love the owner has for their pet. These views mirror the concerns and responses of our participants both in the vignette and in the wider survey.

The second vignette explored views on euthanasia of a dog suffering from an incurable disease. Delayed euthanasia is deemed one of the most prevalent welfare problems for companion animals in the UK (Rioja-Lang *et al.* 2020), and timely euthanasia is considered an important aspect of a veterinarian’s responsibilities (British Veterinary Association 2016). In our vignette, around 40% of UK respondents considered euthanasia of the dog in the baseline scenario to be appropriate and less than 20% disagreed, whereas more than 80% of respondents from Japan showed clear disagreement with this action. Nearly 90% of dogs die through euthanasia in the UK and an average case number for euthanasia per clinic is 5.80 per month (Dickinson *et al.* 2014; Pegram *et al.* 2021), suggesting it to be a common experience for small animal veterinarians. However, in a separate survey, in response to questions regarding whether or not they had conducted euthanasia, over 20% of veterinarians in Japan had not in the previous year, and the average number was 2.48 occasions per clinic per year (Sugita & Irimajiri 2016), suggesting that UK veterinary respondents were much more likely than their Japanese counterparts, to have experience of euthanasia of dogs. A previous interview study reported that veterinarians practising in the UK did not typically regard euthanasia as stressful unless they felt an attachment to the animal, they identified with the owner’s experience or had to deal with convenience euthanasia (O’Connor 2019). A survey conducted in 2019 for practising veterinarians in Japan reported that respondents having not experienced euthanasia found the procedure more stressful than respondents who had experience (Sugita 2022). This suggests that differences in experience might influence how positively euthanasing an animal is viewed.

It has been suggested, under the Aigo concept or with the Buddhism and Shinto cultural background in Japan, that animals should undergo a natural death and not be killed, which could lead to hesitation to euthanase their pets among owners and veterinarians alike (Tsuruta 2012; Sato 2016). In our study, however, a relatively equivalent percentage of Japanese to UK respondents found euthanasia should be performed when the dog’s severe mental stress and pain were apparent. The survey by Sugita and Irimajiri (2016) also reported that 84.9% of veterinarians in Japan agreed to euthanase a companion animal with unrelievable pain or suffering as long as euthanasia is requested by the owner. These results might suggest that Japanese veterinary professionals construe euthanasia as an essential or inevitable procedure for animals that are suffering severely, irrespective of their religious or cultural principles. On the other hand, in the study of Sugita and Irimajiri (2016), only 2.7% of veterinarians agreed to euthanase the animal with incurable suffering when the owner requested further treatment, although an aggressive treatment for terminally ill animals is regarded as a stronger stressor than performing euthanasia for veterinarians in Japan as well as the UK (Batchelor & McKeegan 2012; Sugita 2022). For both countries’ respondents, a request for euthanasia by the owner increased their rate of agreement to euthanase the dog but the change was far more pronounced in the Japanese respondents. These results indicate the possibility that Japan’s much-valued harmonious interdependence between humans (Markus & Kitayama 1991), could manifest as veterinary professionals’ decision-making being substantially influenced by an owner’s opinion regarding the best treatment for their pet. Be it to respect their wishes or even to simply avoid conflict.

Our results using factor analysis suggest that for UK respondents an inability to express normal behaviours, such as eating and walking, was associated with mental suffering as well as severe pain while for Japanese respondents those were independent from mental stress. This alternate perception of the dog’s ability to behave could at least partly explain the lack of accord between the respondents from countries as regards opting for euthanasia when the dog could no longer eat, drink, defaecate, or walk unaided. We suggest that the differing definition of animals – ‘sentient beings’ in UK and ‘living beings’ in Japan – could mirror the cultural attitude toward an animal’s life. In the context of euthanasia of companion animals, the ‘quality of life’ and ‘life worth living’ frameworks are discussed in Western culture (Yeates 2011). With the regard to animals’ sentience, the dog’s life would no longer be worth living once there was an inability to engage in maintenance behaviours as the majority of the UK participants agreed with euthanasia in this case. On the other hand, the ‘living beings’ definition could enable the value or length of an animal’s life to be respected as opposed to their experience and feelings since the Japanese respondents considered it ethically justifiable to keep the dog alive. There have been no studies investigating how the ‘living being’ definition affects attitudes towards understanding for animal welfare, but a Japanese report suggested that a pet’s life being ended by a human decision could be deemed a poorer outcome ethically than waiting for the animal to die naturally with suffering (Sugita 2019). As Yeates (2011) argued, the complexities of quantifying mental experiences of animals and various ethical views regarding the value of an animal’s life have limitations for the ‘life worth living’ concept. The responses to our vignette questions suggest that the thresholds for ‘quality of life’ and ‘a life worth living’ vary between the UK and Japan.

Making the decision to euthanase an animal is highly complex and both owner factors (such as ability to pay, the owners’ perceived benefit of treatment options, owner ability to manage the animal, etc) and animal factors (such as age, individual responses to treatment, relationship with an owner) are relevant. This study used the FF framework to illustrate that contradictory stances on the notion of dog euthanasia held by the UK and Japan might be partially explained by differing perceptions regarding the dog’s ability to express normal behaviours. Asian countries, such as South Korea and Taiwan, are likely to possess a similarly reluctant attitude towards animal euthanasia (Huang *et al.* 2017; UC Davis 2024) while countries with a Western culture, including the US and France, share the same broad attitude that euthanasia for animals is ethically justifiable (Loscos & Marignac 2023). However, each country and culture may show small variations. For instance, only 40% of companion animals had their lives ended by euthanasia in Italy (Pugliese *et al.* 2022), and while Taiwanese law generally prohibits the euthanasia of animals in public shelters, there are no such laws on this in Japan (Yan & Teng 2023). Broadening out this study to incorporate a larger number of countries would provide a deeper insight into cultural variation and how it relates to our understanding of animal welfare.

The limitations of this study include the limited representativeness of the population. Since the survey was distributed via email and social media circulation within several academic associations, those candidates may adhere to stricter professional and ethical values as well as possessing a more profound baseline knowledge of animal welfare compared to non-members. Additionally, the questionnaire only being open online and having participants recruited via social media could have caused systematic bias in the study. For instance, respondents possessing greater familiarity with digital searching may have greater access to information advocating for animal welfare. Also, this online approach may have caused an imbalance in age distribution, especially in the UK. Japan, on the other hand, saw a relatively even balance of respondents. Further, original development of questionnaires did not take content validity into account. This study, however, was the first to develop questions based on the FF and vignettes were carefully created to ensure scenarios were realistic for both countries. Further investigation may be needed to confirm validities, but we believe the findings of our study were valid. It would also be important to note the possible biases of a cross-cultural survey. For instance, it is reported that people in collectivism culture (Japanese respondents in this study) will tend to select an answer toward the centre of a scale or a modest answer whereas people characterised by individualism (as in the UK) are more likely to choose a more extreme answer (Chen *et al.* 1995; Heine *et al.* 2002). However, during questionnaire completion both countries’ respondents were clear in their expressions of agreement/disagreement and the individualism-collectivism theory to select an extreme/centre answer was not observed. Concrete scenarios can be a useful measure of values when comparing the culture (Heine *et al.* 2002). Thus, we conclude our vignettes enabled respondents to think about animal welfare situations more realistically. It is possible that the study participants were influenced by ‘socially desirable’ action for animals in each culture as shown in sections of cat-keeping and dog euthanasia, and people in collectivism are reported to be influenced more than those in individualism (Bernardi 2006). Even if this were the case there was still merit in knowing what factors influenced decision-making on animal management in each country.

## Animal welfare implications and Conclusion

There is no doubt that implementing animal welfare standards is important for all animals in the world. However, as the WOAH describes, animal welfare is a multi-dimensional concept, and encouraging diverse societies and people to make changes to improve animal welfare is challenging. Although the FF describe fundamental principles of animal welfare, a variety of factors, including geography, environment, economy, politics and the cultural perception of animals, sometimes do not allow us to provide all freedoms equally. Recently, a cross-cultural study investigating human perceptions of animals found it important to develop a strategy for providing quality of life to an animal with a respect for diverse cultures (Sinclair *et al.* 2022; Yamanashi *et al.* 2025). This study is the first to compare understanding of FF in different countries and presents novel evidence that veterinarians and animal welfare scientists in the UK and Japan have distinctive attitudes, particularly towards the ‘Freedom to express normal behaviour.’ Further, the results highlighted this difference can lead to alternative interpretations of what constitutes ‘good’ management of animals. This is very relevant when we consider how welfare improvements may be brought about, in both countries, and may suggest conflicts can occur when the ideals of one country are imposed upon another or presented as the only approach to acceptable animal management. Of particular interest in this study is the Japanese concept of Aigo and our data provide preliminary evidence that this might lead to different views from animal welfare regarding what is ethically good in terms of animal management that should be further investigated. So far, as Aigo is officially translated as ‘welfare’ in the English title of the Aigo law (MOE 2014b), there is the possibility that the idea of animal welfare could be interpreted with a different cultural understanding from the UK. Here, our results reconfirmed that there is no one-size-fits-all strategy to implement animal welfare standards and understanding how people perceive animals in different contexts and cultures is extremely important.

## Supporting information

Otani et al. supplementary material 1 Otani et al. supplementary material

Otani et al. supplementary material 2 Otani et al. supplementary material

## Supplementary material

The supplementary material for this article can be found at http://doi.org/10.1017/awf.2025.10026.
