# Supplementary material for: Cross-cultural variation in understanding of animal welfare principles and animal management practices among veterinary and animal welfare professionals in the UK and Japan
Source: Anim Welf. 2025 Aug 6;34:e55. doi: 10.1017/awf.2025.10026 (PMC12451389; doi:10.1017/awf.2025.10026)
Supplement: Otani et al. supplementary material 1 — Otani et al. supplementary material [file S0962728625100262sup001.pdf]

Survey of Attitude towards Animal Welfare in the UK and Japan

This research survey is being undertaken as a collaborative study between the University of Edinburgh in the UK and Hokkaido University in Japan, under the supervision of Professor Cathy Dwyer. Our aim is to investigate views of veterinarians and animal science researchers in the UK and Japan towards animal welfare.

The World Organization for Animal Health (OIE) defines animal welfare is as a complex and multi-faceted subject with scientific, ethical, economic, cultural, social, religious and political dimensions. These different dimensions can lead to quite different types of animal management and views of what is good welfare in different countries. In this project, the same questions in English or in Japanese will be asked to the respondents in the UK and Japan to attempt to understand the differences and similarities, and to investigate effective strategies to improve animal welfare in both countries. This project has received ethical approval from the University of Edinburgh and is partially sponsored by The Japan Society for the Promotion of Science.

The survey typically takes 10-15 minutes and must be completed in one sitting. You will be asked to provide some basic non-identifying information about your history and specialisms. You will then be asked questions related to thinking and understanding of animal welfare.

Your participation in this survey is voluntary. If you feel discomfort or distress whilst answering the questions, you may choose to withdraw your consent without consequence at any time up by closing the Google form page until you click the final "Submit" button. Once you click "Submit" your responses will be fully anonymised and it will not be possible to withdraw. The data we collect do not contain any personal information. Your anonymous data may be used in future publications and presentations relevant to this area of research. The findings of this project will be made available in the website of The Jeanne Marchig International Centre for Animal Welfare Education, The University of Edinburgh.

* Indicates required question

Eligibility of this survey

-Be veterinarians, animal behaviour/welfare researchers, or veterinary science researchers in the UK and Japan
-Live and work in the target countries

Consent Form

By selecting the "I agree" option in the below box, you confirm that:
-You meet the eligibility criteria for this survey.
-You have read the information above and understand the purpose of this survey.
-You understand you are participating voluntarily and may withdraw your consent at any time up until you submit your answers.
-You understand that your responses will be fully anonymous once submitted and as such will not be able to be withdrawn.
-You understand that the data collected will be used in a reports, publications and presentations related to this topic and may also be used by others in future ethically approved research.
-All collected data are protected in accordance with the General Data Protection Regulation (GDPR) 2016/679. https://www.ed.ac.uk/data-protection/data-protection-policy

1. I have read and understood the above information and agree to provide my informed consent to participate in this survey *

Mark only one oval.

- ⬭ Yes
- ⬭ No

Section 1: This section will ask you for some background information about yourself.

2. Where do you currently work? *

Mark only one oval.

- ⬭ UK
- ⬭ Japan

3. What is your age? *

Mark only one oval.

- Under 25
- 25-34
- 35-44
- 45-54
- 55-64
- 65+

4. How do you describe your gender? *

Mark only one oval.

- Female
- Male
- Prefer not to say

5. What is your current profession? *

Mark only one oval.

- veterinarian in practice
- veterinary researcher
- veterinarian in public health
- veterinarian in another role
- researcher of animal behaviour
- researcher of animal welfare
- Other: ____________________

6. What animals are you mainly involved with in your current profession? *

Mark only one oval.

- dogs and cats
- farm animals
- equines
- laboratory animals
- zoo animals
- wildlife
- exotic animals (including small animals except for dogs and cats)
- fish
- I am not involved with animals
- Other: ____________________

Section 2: This section will ask about your basic attitude towards animal welfare and educational history.

7. Do you consider yourself to be familiar with laws or regulations in your country covering animal welfare (or Aigo in Japan)? *

Mark only one oval.

- Strongly agree
- Agree
- Neutral
- Disagree
- Strongly Disagree

8. Have you received any formal education about animal welfare? *

Mark only one oval.

- Yes
- No

9. If you answered yes to the question above, please answer the following: When did you receive animal welfare education? Click all answers that apply.

Tick all that apply.

- [ ] Primary school (-12 years old)
- [ ] High school (12-18 years old)
- [ ] University (undergraduate)
- [ ] University (postgraduate)
- [ ] Continuing professional development
- [ ] Other: ______________________

10. If you received the education in different area from where you currently live, please give the name of the country. [optional]

____

11. Have you undertaken any self-education about animal welfare? *

Mark only one oval.

- ( ) Yes
- ( ) No

12. If you answered yes to the question above, please answer the following: How did you learn it? Click all answers that apply.

Tick all that apply.

- [ ] Reading scientific articles or academic textbooks
- [ ] Attending conferences
- [ ] Massive Open Online Courses (MOOCs)
- [ ] Reading popular science books or articles
- [ ] TV programmes
- [ ] Self-searching for information from relevant organisations (e.g. associations, charities)
- [ ] YouTube
- [ ] Streaming services (e.g. Netflix, Amazon Prime)
- [ ] Other: ______________________

13. Do you think that the citizens in your country (e.g. pet owners) are well educated about animal welfare? *

Mark only one oval.

- Strongly agree
- Agree
- Neutral
- Disagree
- Strongly Disagree

14. Do you want citizens in your country (e.g. pet owners) to be well educated about animal welfare? *

Mark only one oval.

- Strongly agree
- Agree
- Neutral
- Disagree
- Strongly Disagree

Section 3: This section will ask you about your thinking or understanding of the guiding principles of animal welfare 'the five freedoms'.

15. Before taking this survey how many of the Five Freedoms could you explain to other people? *

Mark only one oval.

- 0
- 1
- 2
- 3
- 4
- 5

Please answer the following questions after you know about the five freedoms. You can find definition and information of the five freedoms from the below link of RSCPA.
https://education.rspca.org.uk/documents/1494931/0/FS+The+five+freedoms.pdf/e1e3f7f9-fcce-fdd3-65a8-f29aa4905e2e?t=1555162618511

16. Which of the five freedoms do you think is the most important for companion animals? [Multiple choices allowed. For example if you think some are equally most important then click all answers that apply] *

Tick all that apply.

- [ ] Freedom from hunger, malnutrition and thirst
- [ ] Freedom from fear and distress
- [ ] Freedom from discomfort
- [ ] Freedom from pain, injury and disease
- [ ] Freedom to express normal patterns of behaviour

17. Which of the five freedoms do you think is the most important for farm animals? [Multiple choices allowed. For example if you think some are equally most important then click all answers that apply] *

Tick all that apply.

- [ ] Freedom from hunger, malnutrition and thirst
- [ ] Freedom from fear and distress
- [ ] Freedom from discomfort
- [ ] Freedom from pain, injury and disease
- [ ] Freedom to express normal patterns of behaviour

18. Which of the five freedoms do you think is the most important for experimental animals? [Multiple choices allowed. For example if you think some are equally most important then click all answers that apply] *

Tick all that apply.

- [ ] Freedom from hunger, malnutrition and thirst
- [ ] Freedom from fear and distress
- [ ] Freedom from discomfort
- [ ] Freedom from pain, injury and disease
- [ ] Freedom to express normal patterns of behaviour

19. Which of the five freedoms do you think is the most important for zoo animals? *
[Multiple choices allowed. For example if you think some are equally most important then click all answers that apply]

Tick all that apply.

- [ ] Freedom from hunger, malnutrition and thirst
- [ ] Freedom from fear and distress
- [ ] Freedom from discomfort
- [ ] Freedom from pain, injury and disease
- [ ] Freedom to express normal patterns of behaviour

20. Which of the five freedoms do you think is the most important for wildlife? *
[Multiple choices allowed. For example if you think some are equally most important then click all answers that apply]

Tick all that apply.

- [ ] Freedom from hunger, malnutrition and thirst
- [ ] Freedom from fear and distress
- [ ] Freedom from discomfort
- [ ] Freedom from pain, injury and disease
- [ ] Freedom to express normal patterns of behaviour

Section4: This section will ask you about your thinking on cat welfare using a vignette.

Please read the below vignette and answer the following questions.

*Vignette
• A man who lives alone in an urban area owns one cat.
• The cat is a 2 year old, neutered and microchipped female. She receives regular vaccinations (FPV, FHV, FCV, FeLV) and deworming.
• The owner has a single bedroom flat on the ground floor with access to the outside, surrounded by busy roads.
• The cat is left alone in the flat from 8am to 8pm on weekdays. She has access to cat towers, tunnels and toys indoors.
• The cat is provided with adequate fresh water and food indoors and has access to two litter trays/boxes.
• Feral cats have been seen in the surrounding area.

Please answer the following questions regarding the welfare of this cat. Please note that cats going outside should not have any effect on the security of his flat.

21. How would you recommend that this cat should be kept? *

Mark only one oval.

- Completely indoors
- Mostly indoors, but the cat can go outdoors when she wants.
- Mostly indoors, but the cat can go outdoors when the owner wants.
- Mostly outside, but the cat can go inside when she wants.
- Mostly outside, but the cat can go inside when the owner wants.
- Completely outside

If the cat is kept indoors with access to the outdoors do you think the living conditions allows the cat :

22. access to proper diet and fresh water *

Mark only one oval.

- Strongly agree
- Agree
- Neutral
- Disagree
- Strongly disagree

23. access to somewhere suitable to live *

Mark only one oval.

- Strongly agree
- Agree
- Neutral
- Disagree
- Strongly disagree

24. to interact with other animals *

Mark only one oval.

- Strongly agree
- Agree
- Neutral
- Disagree
- Strongly disagree

25. to express normal behaviour *

Mark only one oval.

- Strongly agree
- Agree
- Neutral
- Disagree
- Strongly disagree

26. to be protected from illness and injury *

Mark only one oval.

- Strongly agree
- Agree
- Neutral
- Disagree
- Strongly disagree

27. to easily be treated for illness and injury *

Mark only one oval.

- Strongly agree
- Agree
- Neutral
- Disagree
- Strongly disagree

28. to avoid conflict with humans *

Mark only one oval.

- Strongly agree
- Agree
- Neutral
- Disagree
- Strongly disagree

29. to avoid becoming lost *

Mark only one oval.

- Strongly agree
- Agree
- Neutral
- Disagree
- Strongly disagree

30. to be deeply loved by the owner *

Mark only one oval.

- Strongly agree
- Agree
- Neutral
- Disagree
- Strongly disagree

If the cat is kept always indoors do you think the living conditions allows the cat:

31. access to proper diet and fresh water *

Mark only one oval.

- Strongly agree
- Agree
- Neutral
- Disagree
- Strongly disagree

32. access to somewhere suitable to live *

Mark only one oval.

- Strongly agree
- Agree
- Neutral
- Disagree
- Strongly disagree

33. to interact with other animals *

Mark only one oval.

- Strongly agree
- Agree
- Neutral
- Disagree
- Strongly disagree

34. to express normal behaviour *

Mark only one oval.

- Strongly agree
- Agree
- Neutral
- Disagree
- Strongly disagree

35. to be protected from illness and injury *

Mark only one oval.

- Strongly agree
- Agree
- Neutral
- Disagree
- Strongly disagree

36. to easily be treated for illness and injury *

Mark only one oval.

- Strongly agree
- Agree
- Neutral
- Disagree
- Strongly disagree

37. to avoid conflict with humans *

Mark only one oval.

- Strongly agree
- Agree
- Neutral
- Disagree
- Strongly disagree

38. to avoid becoming lost *

Mark only one oval.

- Strongly agree
- Agree
- Neutral
- Disagree
- Strongly disagree

39. to be deeply loved by the owner *

Mark only one oval.

- Strongly agree
- Agree
- Neutral
- Disagree
- Strongly disagree

Section 5: This section will ask your thinking about euthanasia using a vignette.

Please read the below vignette and answer the following questions.

*Vignette
• A 9 year old neutered male border collie has been diagnosed with a tumour on his femur, for which there is no hope of a cure.
• The owner is keen to give the dog every possible treatment at home.
• The dog's appetite has decreased to about 50% and the pain is severe, but can be controlled with painkillers at the moment
• The dog is no longer able to run but can walk slowly and still appears to enjoy going outside for a walk.
• He is able to defecate and urinate on his own.

Please consider the following options for the treatment of this dog:

40. Should the dog be euthanised at this point? *

Mark only one oval.

- Strongly agree
- Agree
- Neutral
- Disagree
- Strongly disagree

Under which following conditions would you consider that the dog should be euthanised?

41. When he is no longer able to intake water or food by himself *

Mark only one oval.

- Strongly agree
- Agree
- Neutral
- Disagree
- Strongly disagree

42. When he is no longer able to control defecation and urination *

Mark only one oval.

- Strongly agree
- Agree
- Neutral
- Disagree
- Strongly disagree

43. When the mental stress of the dog becomes severe *

Mark only one oval.

- Strongly agree
- Agree
- Neutral
- Disagree
- Strongly disagree

44. When severe pain can no longer be managed by any medication *

Mark only one oval.

- Strongly agree
- Agree
- Neutral
- Disagree
- Strongly disagree

45. When the dog is no longer able to walk by himself *

Mark only one oval.

- Strongly agree
- Agree
- Neutral
- Disagree
- Strongly disagree

46. When the owner's financial situation worsens *

Mark only one oval.

- Strongly agree
- Agree
- Neutral
- Disagree
- Strongly disagree

47. When the owner physically can no longer care for the dog by himself/herself *

Mark only one oval.

- Strongly agree
- Agree
- Neutral
- Disagree
- Strongly disagree

48. When the owner no longer loves the dog *

Mark only one oval.

- Strongly agree
- Agree
- Neutral
- Disagree
- Strongly disagree

49. When the dog becomes too much trouble for the owner or other humans (e.g. bite, bark) *

Mark only one oval.

- Strongly agree
- Agree
- Neutral
- Disagree
- Strongly disagree

50. When the owner asks vet to euthanise the dog *

Mark only one oval.

- Strongly agree
- Agree
- Neutral
- Disagree
- Strongly disagree

51. Are there other circumstances or responses of the dog where would you recommend to euthanise the dog? [optional]
