# Supplementary material for: Cross-cultural variation in understanding of animal welfare principles and animal management practices among veterinary and animal welfare professionals in the UK and Japan
Source: Anim Welf. 2025 Aug 6;34:e55. doi: 10.1017/awf.2025.10026 (PMC12451389; doi:10.1017/awf.2025.10026)
Supplement: Otani et al. supplementary material 2 — Otani et al. supplementary material [file S0962728625100262sup002.pdf]

Cross-cultural variation in understanding of animal welfare principles and animal management practices among veterinary and animal welfare professionals in the UK and Japan

Yuki Otani https://orcid.org/0000-0003-0224-6197 [1,2,3], Mariko Kanamori[4,5], Hiromi Kato[6], Cathy M Dwyer[1,7]

[1] Jeanne Marchig International Centre for Animal Welfare Education, Royal (Dick) School of Veterinary Studies, University of Edinburgh, Edinburgh, UK

[2] International Affairs Office, Faculty of Veterinary Medicine, Hokkaido University, Sapporo, Japan

[3] Social Cooperation Unit, One Health Research Centre, Hokkaido University, Sapporo, Japan

[4] Institute for the Future of Human Society, Kyoto University, Kyoto, Japan

[5] Department of Public Health Sciences, Stockholm University, Stockholm, Sweden

[6] Animal Welfare and Wildlife Damage Management Group, National Agriculture and Food Research Organisation, Tsukuba, Japan

[7] Animal Behaviour and Welfare, Department of Animal and Veterinary Sciences, Central Faculty, Scotland's Rural College (SRUC), Edinburgh, UK

Author for correspondence: Yuki Otani, email: yukiotani35@vetmed.hokudai.ac.jp

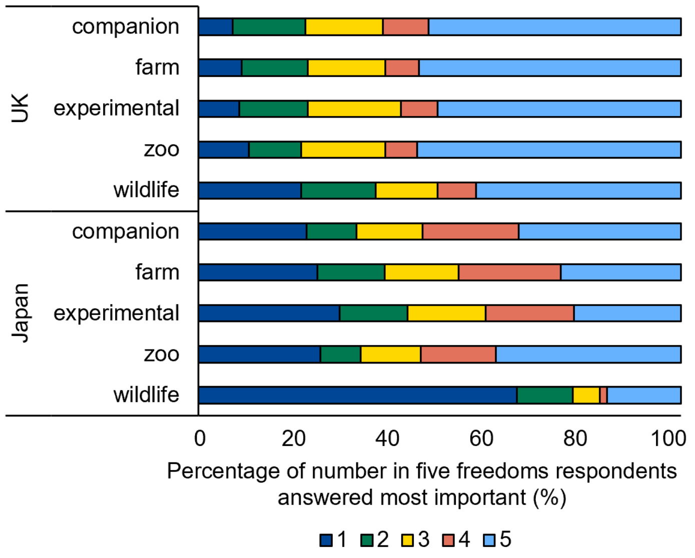

Figure S1. Bar graph of the percentage of UK (upper panel; n = 212) and Japanese (lower panel; n=321) respondents who selected 1, 2, 3, 4, or 5 freedoms as the most important (or equally important) of the Five Freedoms for five different categories of animal (companion, farm, experimental, zoo and wildlife).

Table S1. Result of binary logistic regression using demographic data to investigate predictors of receiving an education about animal welfare for veterinary professionals and animal welfare researchers in the UK (n = 212) and Japan (n = 321). Younger respondents had more formal education about animal welfare in Japan whereas no generation difference was observed in the UK. For self-education, more animal welfare researchers had sought to find education themselves than veterinarians.

	**Formal-education**						**Self-education**					
	**UK**			**JAPAN**			**UK**			**JAPAN**		
	OR	*P*	[95% CI]	OR	*P*	[95% CI]	OR	*P*	[95% CI]	OR	*P*	[95% CI]
**Age (ref. <35)**												
35-44	2.89	0.34	[0.33, 24.95]	0.36	0.007	[0.18, 0.76]	1.58	0.21	[0.77, 3.23]	0.78	0.42	[0.42, 1.43]
45-54	0.24	0.07	[0.52, 1.12]	0.07	<0.001	[0.32, 0.15]	1.94	0.29	[0.57, 6.54]	0.89	0.74	[0.46, 1.74]
55<	0.32	0.17	[0.062, 1.61]	0.39	<0.001	[0.016, 0.096]	6.32	0.02	[1.34, 29.78]	1.38	0.41	[0.64, 2.96]
**Profession (ref. Vet in practice)**												
Veterinary researcher	1.4	0.67	[0.30, 6.46]	2.64	0.002	[1.43, 4.88]	1.47	0.37	[0.63, 3.40]	1.78	0.034	[1.05, 3.04]
Animal behaviour/welfare researcher	2.12	0.5	[0.24, 18.73]	1.55	0.39	[0.58, 4.13]	7.24	0.009	[1.63, 32.16]	15.53	0.008	[1.05, 117.50]

OR: Odds Ratio; CI: Confidence Interval

Table S2. Results of Chi-square test between responses of veterinary professionals in the UK (n = 212) and Japan (n = 321) to a survey of understanding and attitudes to the Five Freedoms. The data show the comparison of proportions of respondents who thought a freedom was most important in the Five Freedoms for each of five categories of animal use. For companion, farm, experimental and zoo animals, 'Freedom from injury and disease' and 'Freedom to express normal behaviour' were less frequently selected by respondents in Japan compared to respondents in the UK although the percentage of respondents who selected the 'Freedom to express normal behaviour' for wildlife was equivalent between countries.

		Hunger and Thirst	Fear and Distress	Discomfort	Pain, Injury and Disease	Express Normal Behaviour
**Companion**	$\chi2(1)$	3.28	2.35	0.35	22.52	35.99
	P	0.07	0.13	0.55	< 0.001	< 0.001
**Farm**	$\chi2(1)$	8.19	7.20	< 0.001	36.78	56.33
	P	0.00	0.01	0.99	< 0.001	< 0.001
**Experimental**	$\chi2(1)$	6.38	12.58	3.80	26.65	65.70
	P	0.01	<0.001	0.05	< 0.001	< 0.001
**Zoo**	$\chi2(1)$	6.69	7.06	0.35	22.90	13.38
	P	0.01	0.01	0.55	< 0.001	< 0.001
**Wildlife**	$\chi2(1)$	82.12	72.57	42.63	123.08	3.37
	P	< 0.001	< 0.001	< 0.001	< 0.001	0.07
